# Immune pressures drive the promoter hypermethylation of neoantigen genes

**DOI:** 10.1186/s40164-019-0156-7

**Published:** 2019-11-29

**Authors:** Ming Yi, Bing Dong, Qian Chu, Kongming Wu

**Affiliations:** 1 Department of Oncology, Tongji Hospital of Tongji Medical College, Huazhong University of Science and Technology, Wuhan, 430030 China; 2 Department of Molecular Pathology, The Affiliated Cancer Hospital of Zhengzhou University & Henan Cancer Hospital, Zhengzhou, 450008 China

**Keywords:** Immunoediting, Neoantigens, Immune evasion, Hypermethylation, Epigenetics

## Abstract

Cancer cells with strong immunogenicity are susceptible for elimination by cancer immunoediting, while the subpopulations with weak immunogenicity survive. As a result, a subset of cancer cells evade the immune attack and evolve into overt clinical lesions. During cancer evolution, it has been well established that multiple alterations such as the dysfunction of antigen presentation machinery and the upregulation of immunosuppressive signals (e.g. PD-L1) play important roles in immune escape. Recently, promoter hypermethylation of neoantigen genes has been proposed to be a vital mechanism of immunoediting. This epigenetically mediated immune evasion enriches the mechanisms of carcinogenesis.

Neoantigens arise from somatic mutations and are exclusively expressed in cancer cells. These tumor-associated antigens are the ideal targets for immune recognition and attack. Derived by immune pressures, cancer cells down-regulate the recognizable targets on their surfaces and evolve into weakly immunogenic subclones [1]. It is generally believed that the loss of complex formation between neopeptide and major histocompatibility complex (MHC) in cancer cells is responsible for the acquired dysfunction of antigen processing and presentation [2].

Recently, Rosenthal et al. found that the hypermethylation of the promoter of neoantigen genes participated in the decreased cancer immunogenicity [3]. In this study, Rosenthal et al. analyzed immune infiltration statuses of untreated non-small cell lung cancer (NSCLC) patients by RNA-sequencing and tumor infiltrating lymphocyte (TIL) histopathology estimates [3]. The study showed that just 33% clonal neoantigens were ubiquitously expressed in every region of a given tumor [3]. Further investigation revealed that the proportion of ubiquitously expressed clonal neoantigens was significantly decreased in tumors with abundant TILs compared to tumors with scarce TILs (41% vs. 29%, P = 0.01) [3]. At the transcription level, the researchers observed immune pressure-caused neoantigen depletions [3]. Using the multi-region reduced representation bisulfite sequencing, it was detected that the genes carrying neoantigenic mutations harbored 11.4-fold increase in hypermethylation of promoters when compared to other genes (P = 0.00016) [3]. To verify whether this increased hypermethylation was neoantigen-specific or not, the researchers compare the methylation statuses between neoantigens and corresponding wild type genes. The results indicated that these non-expressed neoantigens were more likely to possess increased promoter methylation (odds ratio = 2.33, P = 0.045) [3].

These findings demonstrated that the neoantigen silencing was the result of immune pressures via promoter hypermethylation. The loss of neoantigens is a core event of immunoediting and immune evasion. Abundant neoantigens released from cancer cells initiate robust anti-cancer immune responses [4]. Then, professional antigen presentation cells (APCs) take in and process these neoantigens [4]. Subsequently, in peripheral lymphoid organs, the naïve T lymphocytes are primed and activated by APCs [4]. These activated T cells could migrate and infiltrate into tumors. Eventually, TILs recognize and kill cancer cells [4]. As a result, the release of more neoantigens propagate the anti-cancer immune response [4]. It is well accepted that cancer cells can adopt multiple manners to counteract immune clearance such as secreting anti-inflammation cytokines, upregulating immune checkpoint signals, counter-attacking TILs via increasing Fas ligand (Fas-L) expression, and disabling antigen presentation machinery (Fig. 1) [5, 6]. As the hallmark of cancer cells, neoantigens are generated as the by-products of accumulated somatic mutations [7]. Theoretically, tumor-associated neoantigens are ideal targets for immunotherapies with chimeric antigen receptor T cells (CAR-T) and bi-specific antibodies [8, 9], though in reality, resistance to these cancer neoantigen-targeted immunotherapies still remains a major challenge [10]. The results of Rosenthal et al. provide a novel perspective to the understanding of carcinogenesis and cancer evolution under immune pressure. Moreover, this study suggests that combination of hypomethylating agents with immunotherapy might offer double attack on neoantigen-rich cancers.

**Fig. 1:**
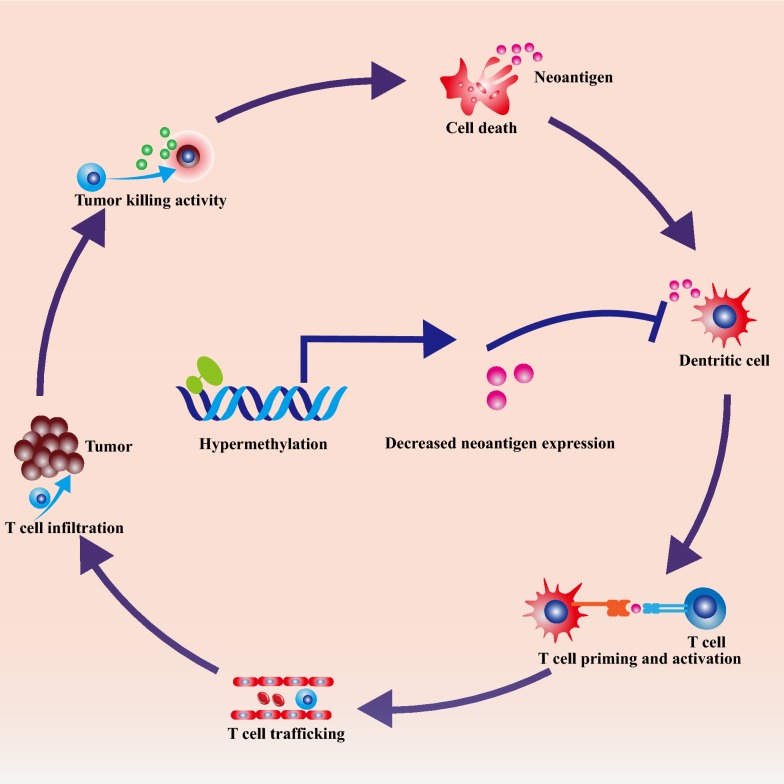
Promoter hypermethylation-mediated neoantigen downregulation leads to evasion of cancer immune response. Release of abundant neoantigens initiate anti-cancer immune response. Then, professional antigen presentation cells (APCs) take in and process these neoantigens. Subsequently, in peripheral lymphoid organs, the naïve T lymphocytes are primed and activated by APCs. These activated T cells migrate and infiltrate into tumors (TILs). These TILs recognize and destroy cancer cells. As a result, more neoantigens propagate the anti-cancer immune response. Under these immune pressure, cancer cells downregulate neoantigen expression by promoter hypermethylation and evolve into weakly immunogenic subclones

## Data Availability

Data sharing not applicable to this article as no datasets were generated or analyzed during the current study.

## Abbreviations

MHC: major histocompatibility complex
NSCLC: non-small cell lung cancer
TIL: tumor infiltrating lymphocyte
APC: antigen presentation cell
Fas-L: Fas ligand
CAR-T: chimeric antigen receptor T cell
